# A biochemical logarithmic sensor with broad dynamic range

**DOI:** 10.12688/f1000research.14016.3

**Published:** 2018-04-18

**Authors:** Steven A. Frank

**Affiliations:** 1 Department of Ecology and Evolutionary Biology, University of California, Irvine, CA, 92697-2525, USA

**Keywords:** Biochemical circuit, Hill equation, synthetic biology, systems biology, aggregation, robustness

## Abstract

Sensory perception often scales logarithmically with the input level. Similarly, the output response of biochemical systems sometimes scales logarithmically with the input signal that drives the system. How biochemical systems achieve logarithmic sensing remains an open puzzle. This article shows how a biochemical logarithmic sensor can be constructed from the most basic principles of chemical reactions. Assuming that reactions follow the classic Michaelis-Menten kinetics of mass action or the more generalized and commonly observed Hill equation response, the summed output of several simple reactions with different sensitivities to the input will often give an aggregate output response that logarithmically transforms the input. The logarithmic response is robust to stochastic fluctuations in parameter values. This model emphasizes the simplicity and robustness by which aggregate chemical circuits composed of sloppy components can achieve precise response characteristics. Both natural and synthetic designs gain from the power of this aggregate approach.

## Introduction

I present a simple biochemical circuit that logarithmically transforms input signals. This circuit adds the outputs of several reactions that follow standard mass action Michaelis-Menten kinetics. Alternatively, the biochemical kinetics may follow the commonly observed Hill equation response, which includes Michaelis-Menten kinetics as a special case. This sensor has high dynamic range, responding logarithmically across many orders of magnitude. The high dynamic range is achieved by adding together reactions with different sensitivity ranges. The aggregate nature of this circuit provides robustness to parameter variations. Aggregate sensor design may explain the commonly observed high dynamic range of logarithmic biological responses and may also provide a useful tool for synthetic biology.

## Results and discussion

Many biochemical reactions and cellular responses transform an input, *x*, into an output, *y*, according to the Hill equation

$$y=\frac{x^{k}}{c^{k}+x^{k}},$$

in which *c* is the value of the input *x* that yields one-half of the maximal output response, *k* is the Hill coefficient that determines the shape of the response, and *y* is normalized to be between 0 and 1 ^1^. Simple mass action kinetics often follows the Hill equation with *k* = 1, which corresponds to classical Michaelis-Menten kinetics ^2^. For example, the input may drive production of the output, and the output may spontaneously transform back to a prior state.

**Figure 1.**
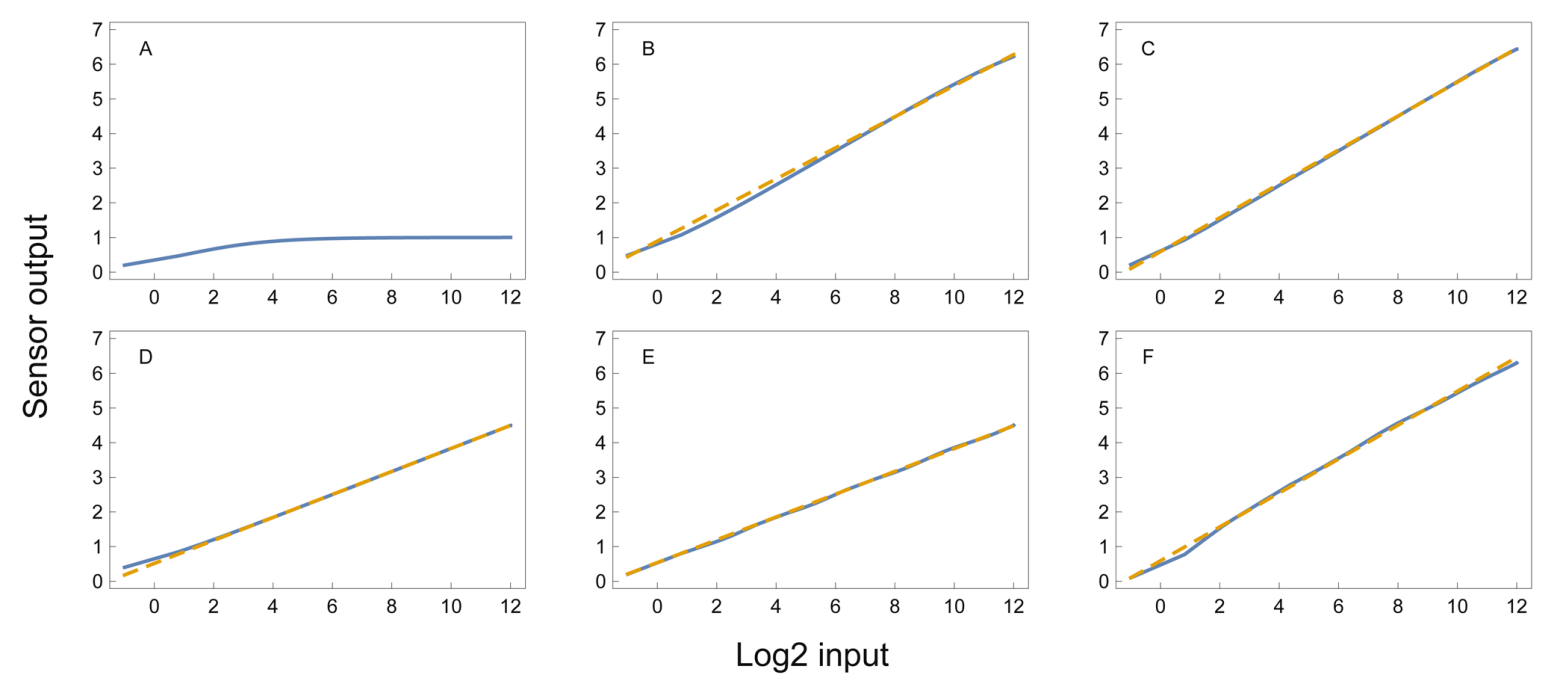
The logarithmic sensor response, *y*, from the sum of Hill equations given in Equation 1, versus the logarithm of the input, *x*, on a log _2_ scale. Each unit on the input scale corresponds to a doubling of the input. The range of 13 doublings is approximately four orders of magnitude, 2 ^13^ ≈ 10 ^4^. A logarithmic sensor responds linearly with respect to the logarithm of the input. The solid blue lines show the response, *y*, from Equation 1. The dashed gold lines are linear fits to the response. ( **A**) A single Hill equation with *c* = 1 and *k* = 2. A linear response with low sensitivity occurs over a few doublings at small input levels. The following plots all use *n* = 7 and additional parameters as described from the summed Hill equation in Equation 1, in which *c _i_* = *b ^i^*. ( **B**) Response with *k* = 1 and *b* = 4. ( **C**) Response with *k* = 2 and *b* = 4. ( **D**) Response with *k* = 1 and *b* = 8. ( **E**) Response with *k* = 2 and *b* = 8. ( **F**) Same average parameters values of *k* = 2 and *c _i_* = *b ^i^* for *b* = 4 as in the plot above, but with random parameter fluctuations around those average values. For each of the 7 Hill equations in the sum given in Equation 1, each parameter was obtained by a different random number drawn from a normal distribution. For each *k*, the parameters were drawn from a normal distribution with mean 2 and standard deviation 2 × 0.25 = 0.5. For each *c _i_*, the parameters were drawn from a normal distribution with mean 4 *^i^* and standard deviation 4 *^i^* ×0.25. The response remains nearly linear in spite of the random parameter fluctuations (see Data availability).

The output of the Hill equation scales approximately logarithmically with its input through the middle part of its response range, because *y* is roughly linear with respect to log *x*. Prior studies have emphasized that a Hill equation response can act as a logarithmic sensor ^3, 4^. However, a single Hill equation response provides a logarithmic sensor with limited dynamic range ( Figure 1A).

My extended dynamic range sensor arises by adding together *n* Hill equations with increasing values of the half-maximal response, *c _i_*, as

$$y=\sum_{i=0}^{n-1}\frac{x^{k}}{c_{i}^{k}+x^{k}}.\;(1)$$

**Figure 2.**
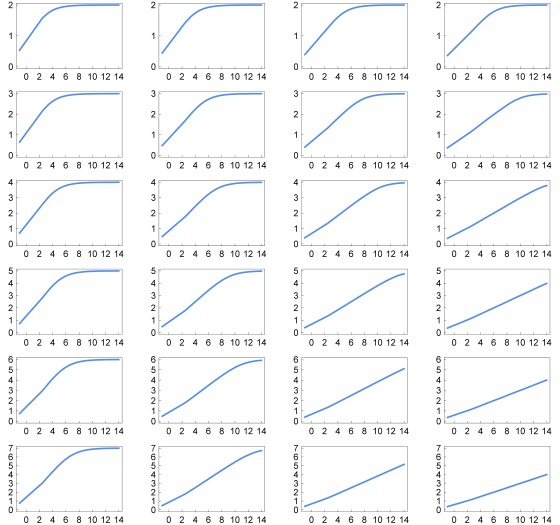
The logarithmic sensor response as in Figure 1, with the rows from top to bottom showing *n* = 1,2,3,4,5,6 and the columns from left to right showing *b* = 2,4,8,16. Note that the scale along the *x*-axis is increased to 2 ^14^ compared with 2 ^12^ in Figure 1. All plots use the Michaelis-Menten relation with Hill equation coefficient *k* = 1 (see Data availability).

**Figure 3.**
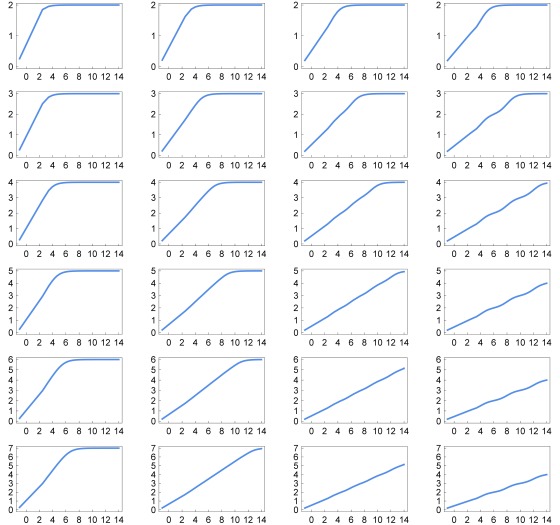
The logarithmic sensor response as in Figure 2, with Hill equation coefficient *k* = 2 (see Data availability).

For example, if *c _i_* = *b ^i^*, then each reaction in the sum has an increasing input value at which its maximal sensitivity occurs. Figure 1B–E shows that simple combinations of *k* and *b* create a logarithmic sensor, in which the output is linearly related to the logarithm of the input. The logarithmic relation holds robustly when the parameters *k* and *b* of the individual reactions vary stochastically ( Figure 1F). Figure 2 and Figure 3 show the response for various parameter combinations.

Several biochemical circuit responses and many aspects of perception scale logarithmically ^5^. A robust and generic pattern of this sort seems likely to depend on a robust and generic underlying design. In the search for a generic circuit, my biochemical logarithmic sensor has three advantages over prior designs. First, prior models depended on particular molecular assumptions about biochemical kinetics or reaction pathways ^3, 4^. My design requires only Michaelis-Menten or Hill equation responses, which are very widely observed in biochemical and cellular systems ^1^. Second, prior models focused on single input-output processes, which have relatively narrow dynamic range. My aggregate design provides a logarithmic response over a vastly greater range. Third, the prior models’ responses are easily perturbed by parameter fluctuations. My design performs robustly with respect to broad fluctuations in parameters. The robustness of my logarithmic sensor emphasizes the potential to achieve precise response characteristics from underlying sloppy components when using an aggregate design ^6, 7^.

## Data availability

Mathematica source code to produce Figure 1 can be found at: https://doi.org/10.5281/zenodo.1217658 ^8^

License: CC BY 4.0
