# Supplementary material for: A biochemical logarithmic sensor with broad dynamic range
Source: F1000Res. 2018 Apr 18;7:200. Originally published 2018 Feb 16. [Version 3] doi: 10.12688/f1000research.14016.3 (PMC5897783; doi:10.12688/f1000research.14016.3)
Supplement: Peer review history

# Peer review: Referee response for version 2

**DOI:** 10.5256/f1000research.15653.r32535

**Published:** 2018-04-11

**Authors:** Marco Archetti

**Affiliations:** 1 School of Biological Sciences, University of East Anglia, Norwich, UK

This is a brilliant short paper: a clever idea, explained very clearly and that immediately makes sense (but that is by no means obvious at first sight). The revision and the comments about the first version seem appropriate to me. 

 I don’t have further substantial comments except perhaps the following. 

 I was curious to understand what value of *n* is required to achieve the result presented in the figure (is there anything special about *n*=7?). Using the code provided this is easy to check, but perhaps it would help readers to add an additional figure with multiple curves for values of *n* ranging between 1 and 7 to see how the results change. Doing this exercise myself I learned, for instance, that *n*<7 is enough for certain parameters and that the value of *b* is especially important (something that perhaps could be discussed in a sentence or two).

I have read this submission. I believe that I have an appropriate level of expertise to confirm that it is of an acceptable scientific standard.

# Response

**Published:** 2018-04-12

**Authors:** Steven Frank

**Affiliations:** Department of Ecology & Evolutionary Biology, University of California, Irvine, USA

I thank Dr. Archetti for his suggestion to illustrate the response over various values of the parameters *n* and *b*. In the new revision of the article, I have added Figures 2 and 3 to show the sensor's response over a broad range of these parameters.

# Peer review: Referee response for version 2

**DOI:** 10.5256/f1000research.15653.r32373

**Published:** 2018-04-04

**Authors:** Sudin Bhattacharya

**Affiliations:** 1 Department of Biomedical Engineering, Department of Pharmacology & Toxicology, Institute for Quantitative Health Science and Engineering, Michigan State University, East Lansing, MI, USA

I am satisfied with the author's response to my comments about his article, which at this stage is a theoretical model of how a natural or artificial biochemical logarithmic sensor *might* work.

I have read this submission. I believe that I have an appropriate level of expertise to confirm that it is of an acceptable scientific standard.

# Peer review: Referee response for version 1

**DOI:** 10.5256/f1000research.15234.r30929

**Published:** 2018-03-19

**Authors:** Sudin Bhattacharya

**Affiliations:** 1 Department of Biomedical Engineering, Department of Pharmacology & Toxicology, Institute for Quantitative Health Science and Engineering, Michigan State University, East Lansing, MI, USA

The author presents an interesting model of a robust biochemical logarithmic switch with a broad dynamical range as a sum of multiple Hill-equation based models.

1. My main problem with this work is the lack of a biological context with a real example (the author argues the model applies to natural as well as artificial circuits). Is the author proposing that the successive Hill-type switches would be arranged in series? A more natural example would be a layered cascade like a MAP kinase cascade ^1^. But in that case the output would not be a simple linear sum as in the author’s Eqn. (1), but rather something like a Hill function of a Hill function.
2. The contention that “My design performs (more) robustly with respect to broad fluctuations in parameters” should be established more rigorously. How were the stochastic simulations performed? It’s hard to tell since no methodological details are provided.
3. “Menten” in Michaelis-Menten mis-spelt throughout manuscript (after Maud Menten: https://en.wikipedia.org/wiki/Maud_Menten)

I have read this submission. I believe that I have an appropriate level of expertise to confirm that it is of an acceptable scientific standard, however I have significant reservations, as outlined above.

## References

[1] HuangCYFerrelJE: Ultrasensitivity in the mitogen-activated protein kinase cascade. *Proceedings of the National Academy of Sciences*.1996;93:10078-10083 10.1073/pnas.93.19.10078PMC383398816754

# Response

**Published:** 2018-03-19

**Authors:** Steven Frank

**Affiliations:** Department of Ecology & Evolutionary Biology, University of California, Irvine, USA

I thank Dr. Bhattacharya for sharing his expertise on topics related to the important Hill equation (see ref. 1 of the article). Dr. Bhattacharya made three helpful comments in his review, which I respond to in reverse order.

 First, I appreciate the spelling correction. I had reread the interesting history of the Michaelis-Menten equation when preparing this article, but nonetheless incorrectly spelled Menten's name. I have fixed that misspelling in the revision.

 Second, I have uploaded the source code, as a supplementary file, that I used to do the stochastic simulations. The short, simple code provides a clear, exact description of the methods. The code was used to generate Figure 1, and can be used to regenerate that figure and explore alternative assumptions. A summary of the methods are provided in the legend for Figure 1.

 The final point concerns the lack of specific biological examples and details. The first aspect of this comment is a question asked by Dr. Bhattacharya: "Is the author proposing that the successive Hill-type switches would be arranged in series?"

 I am proposing a summation of individual responses to a stimulus, as described in Equation 1. For example, suppose that a set of sensors respond to sound intensity. Each sensor has a Hill-like response curve, but tuned to different intensity level. The output of the aggregate set of sensors is the sum of the individual sensor responses. Each sensor may comprise a simple or complex set of reactions. All that we care about here is that the net response per sensor is Hill-like in shape.

 That same logic could describe the response to a molecular signal. Various reactions could each have a Hill equation response, but tuned to different sensitivities. The aggregate consequence of those responses would be a net output level.

 With regard to an explicit example, I gave a lot of thought to that issue while preparing the manuscript. I could not find a compelling example for which sufficient biological detail about mechanism had been described. There are plausible cases that are worth exploring, such as the sensory problems that I mentioned. For molecular systems, there are hormones for which cells have multiple receptors, presumably tuned differently with regard to response and other aspects (e.g., estrogen receptors). However, I agree that those multireceptor systems do not provide a compelling case given current knowledge.

 Why are there no compelling molecular examples? I think the reason is that researchers rarely look for responses that arise by aggregate processes, such as summation of underlying reactions. Instead, the search is almost always for a single reaction or explicit pathway that exactly matches the desired input-output pattern. So my goal for this article is to suggest a plausible alternative for consideration.

 One advantage of publishing with F1000Research is that I can update the article in the future. If a good example arises in discussion with readers of the article or from the literature, I will update the article to include that example. For now, I think it is most important that the novel idea be published in a concise and direct manner, with emphasis on the simple logic of the model.
